# Metformin enhances TRAIL-induced apoptosis by Mcl-1 degradation *via* Mule in colorectal cancer cells

**DOI:** 10.18632/oncotarget.11147

**Published:** 2016-08-09

**Authors:** Seong Hye Park, Dae-Hee Lee, Jung Lim Kim, Bo Ram Kim, Yoo Jin Na, Min Jee Jo, Yoon A. Jeong, Suk-Young Lee, Sun Il Lee, Yong Yook Lee, Sang Cheul Oh

**Affiliations:** ^1^ Brain Korea 21 Program for Biomedicine Science, Korea University College of Medicine, Korea University, Seoul, Republic of Korea; ^2^ Division of Oncology/Hematology, Department of Internal Medicine, Korea University College of Medicine, Seoul, Republic of Korea; ^3^ Department of Surgery, Korea University Guro Hospital, Korea University College of Medicine, Seoul, Republic of Korea; ^4^ The Korean Ginseng Research Institute, Daejeon, Republic of Korea

**Keywords:** metformin, TRAIL, CRC, Mcl-1, Noxa

## Abstract

Metformin is an anti-diabetic drug with a promising anti-cancer potential. In this study, we show that subtoxic doses of metformin effectively sensitize human colorectal cancer (CRC) cells to tumor necrosis factor (TNF)-related apoptosis-inducing ligand (TRAIL), which induces apoptosis. Metformin alone did not induce apoptosis, but significantly potentiated TRAIL-induced apoptosis in CRC cells. CRC cells treated with metformin and TRAIL showed activation of the intrinsic and extrinsic pathways of caspase activation. We attempted to elucidate the underlying mechanism, and found that metformin significantly reduced the protein levels of myeloid cell leukemia 1 (Mcl-1) in CRC cells and, the overexpression of Mcl-1 inhibited cell death induced by metformin and/or TRAIL. Further experiments revealed that metformin did not affect mRNA levels, but increased proteasomal degradation and protein stability of Mcl-1. Knockdown of Mule triggered a significant decrease of Mcl-1 polyubiquitination. Metformin caused the dissociation of Noxa from Mcl-1, which allowed the binding of the BH3-containing ubiquitin ligase Mule followed by Mcl-1ubiquitination and degradation. The metformin-induced degradation of Mcl-1 required E3 ligase Mule, which is responsible for the polyubiquitination of Mcl-1. Our study is the first report indicating that metformin enhances TRAIL-induced apoptosis through Noxa and favors the interaction between Mcl-1 and Mule, which consequently affects Mcl-1 ubiquitination.

## INTRODUCTION

Colorectal cancer (CRC), both the third most common form of cancer and the third leading cause of cancer-related death worldwide, can attribute its effect in part to the metastases of its parent tumor [1]. Numerous studies have recently made remarkable advances in the search for a cure for metastatic CRC. For instance, the addition of irinotecan and oxaliplatin to 5-fluorouracil (5-FU) increased the median overall survival of 1 year [2]. Median overall survival was also increased by nearly a factor of two to 20 months when monoclonal antibody therapies such as cetuximab and bevacizumab were used [3–5]. Despite these advances, there remains a lack of effective drugs to improve overall survival in patients with CRC. Therefore, development of new therapies for metastatic CRC is urgent.

Tumor necrosis factor (TNF)-related apoptosis-inducing ligand (TRAIL), a death ligand and a promising anticancer drug, is a member of the TNF-α superfamily that binds to the death receptors TRAIL-R1 (DR4) [6, 7] and TRAIL-R2 (DR5) [8, 9]. While TRAIL generally does not induce normal primary cell death, TRAIL-mediated death of tumor cells occurs via extrinsic and intrinsic cell-death signaling pathways [10]. In the extrinsic pathway, DR-mediated apoptosis occurs when TRAIL binds to DR-formed trimers of the TRAIL receptors, adaptor proteins, Fas-associated protein with death domain (FADD), and caspase-8, to form the death-inducing signaling complex (DISC) during cell death. The activation of caspase-8 leads quickly to the formation of caspase-3 (activation form), which consequently induces cancer cell apoptosis. The intrinsic pathway is also called the mitochondria-dependent apoptosis pathway. Previous studies show that TRAIL-induced cell death can be promoted by combinational treatment with various anticancer drugs at minimal toxic doses in tumor cells [11–13]. Recent reports, however, indicate that various types of cancers are resistant to TRAIL [14]. Therefore, treatment with TRAIL alone may be insufficient to overcome resistance. Thus, understanding the roles that TRAIL play and the discovery of effective sensitizers for TRAIL-mediated cancer therapy are the major challenges for the development of novel therapeutic strategies for cancer treatment.

While promising results have been reported in Chinese phase III clinical trials in which TRAIL has been used as an anti-cancer therapy, resistance to TRAIL-induced apoptosis remains a main obstacle in its future clinical application. Many recent reports have focused on improving TRAIL sensitization as a way to mediate tumor cell death. Using this strategy, several novel anticancer drugs that are capable of increasing sensitization to TRAIL have been developed. For instance, wogonin, capsaicin, verrucarin A, curcumin, epigallocatechin gallate, and resveratrol, among others, have been reported to sensitize tumor cells to TRAIL-induced cell death, mostly through the cell surface receptor pathway via up-regulation of DR5/DR4 through c-FLIP [15–20]. Studies have also showed that TRAIL-induced cell death can be regulated via the intrinsic pathway. By inhibiting two anti-apoptotic proteins, Bcl-2 and Bcl-XL, or by increasing the expression of pro-apoptotic proteins Bad, Bax, Bim, and Bcl-xs, the sensitivity of many cancers to TRAIL treatments can be enhanced [20].

In addition, resistance to TRAIL among different types of cancer poses a major obstacle to effective therapy [21, 22]. Therefore, scientists are in search for compounds that sensitize tumor cells to TRAIL and reverse resistance in tumor cells. This line of inquiry is important because, while approximately 60% of human tumors have been found to be TRAIL-resistant, the mechanism of resistance is poorly understood [23–26].

**Figure 1:**
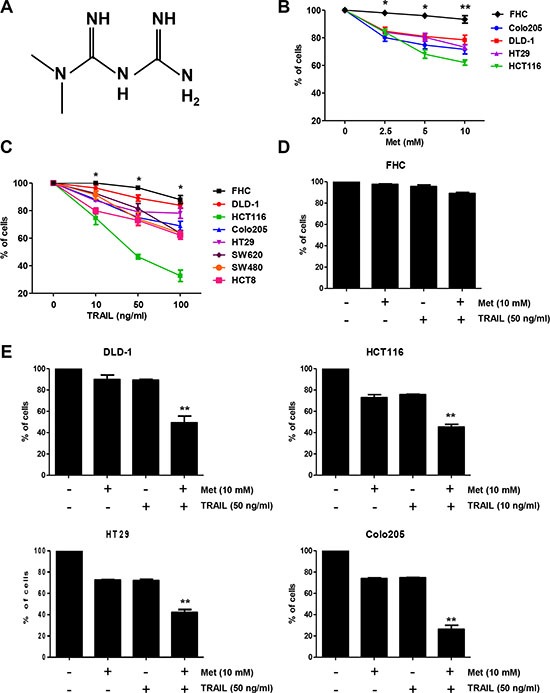
Metformin significantly increased TRAIL-induced cytotoxicity of human CRC cell lines (**A**) The structure of metformin. Effects of metformin (**B**) and TRAIL (**C**) alone, or a combination treatment of metformin and TRAIL (**D**, **E**) on cytotoxicity of human CRC cell lines (mean ± SD, *n* = 3). Cells were treated with DMSO (sham control) or various concentrations (0–10 mM) of metformin for 20 h. Cells were incubated in the presence or absence of TRAIL (10 or 50 ng/ml) and/or metformin (10 mM) for 24 h. Experiments were performed at least three times. Error bars represent standard error of the mean (SEM) from three separate experiments. Some error bars are too small to be seen. Asterisk * or ** represents a statistically significant difference between FHC and cancer cells at *p* < 0.05 or *p* < 0.01, respectively.

In the current study, we sought to assess the possible sensitizing effect of metformin on TRAIL-mediated cell death in CRC cells. In recent studies, diabetic patients receiving metformin (Figure 1A), a commonly used medication to lower blood glucose [27, 28], had decreased cancer mortality and a reduced risk of developing cancer [29–32]. Moreover, metformin sensitizes human cancer cells to classical anti-cancer agents, such as dasatinib, paclitaxel, and aspirin [33–35]. In preclinical studies, metformin inhibited proliferation, invasion, and migration of pancreatic cancer cells [36, 37] while it improved the survival outcomes of patients with CRC and diabetes in previous studies [38]. Notably, for the first time, our data shows that combining TRAIL and low doses of nonapoptosis-inducing metformin enhance apoptosis in human CRC cell lines. We also present the first evidence that metformin upregulates Bax and downregulates Mcl-1 as well as synergistically increases TRAIL-mediated cell death in human CRC cell lines.

## RESULTS

### Combination of metformin and TRAIL synergistically enhances CRC cell death, but not that of normal primary colon cells

Metformin has been previously reported to induce apoptosis in several cell types such as human cervical cancer, human ovarian, human leukemia, and human CRC [39–42]. Before investigating the effect of combined treatment with metformin and TRAIL on viability of CRC cells, we evaluated whether metformin alone induces cell death. CRC cells were exposed to 2.5–10 mM metformin for 24 h. Here, we found that metformin-induced cell death in a dose-dependent manner (Figure 1B). Cancer cell lines displayed various levels of sensitivity, but normal primary colon cells (FHC) were resistant to the drug. In normal colorectal cells, minimal cytotoxicity (7% killing) was observed at a high dose (10 mM) of metformin, while in the CRC cell lines, sensitivity was observed even at 2.5 mM. The effect of treatment with a combination of metformin and TRAIL was investigated in several CRC cell lines as well as FHC cells. Cytotoxicity was induced by TRAIL alone, in FHC cells in a dose-dependent manner (Figure 1C). Cytotoxicity was significantly enhanced by combined treatment with metformin and TRAIL in TRAIL-sensitive HCT116 cells and TRAIL-resistant DLD-1, HT29, and Colo205 cells (Figure 1E), but not in normal primary colon cells (FHC) (Figure 1D). These results suggest that the sensitizing regimen of metformin plus TRAIL may be selectively toxic to CRC cells.

### Metformin facilitates TRAIL-induced apoptosis in CRC cells through activation of extrinsic and intrinsic pathway

**Figure 2:**
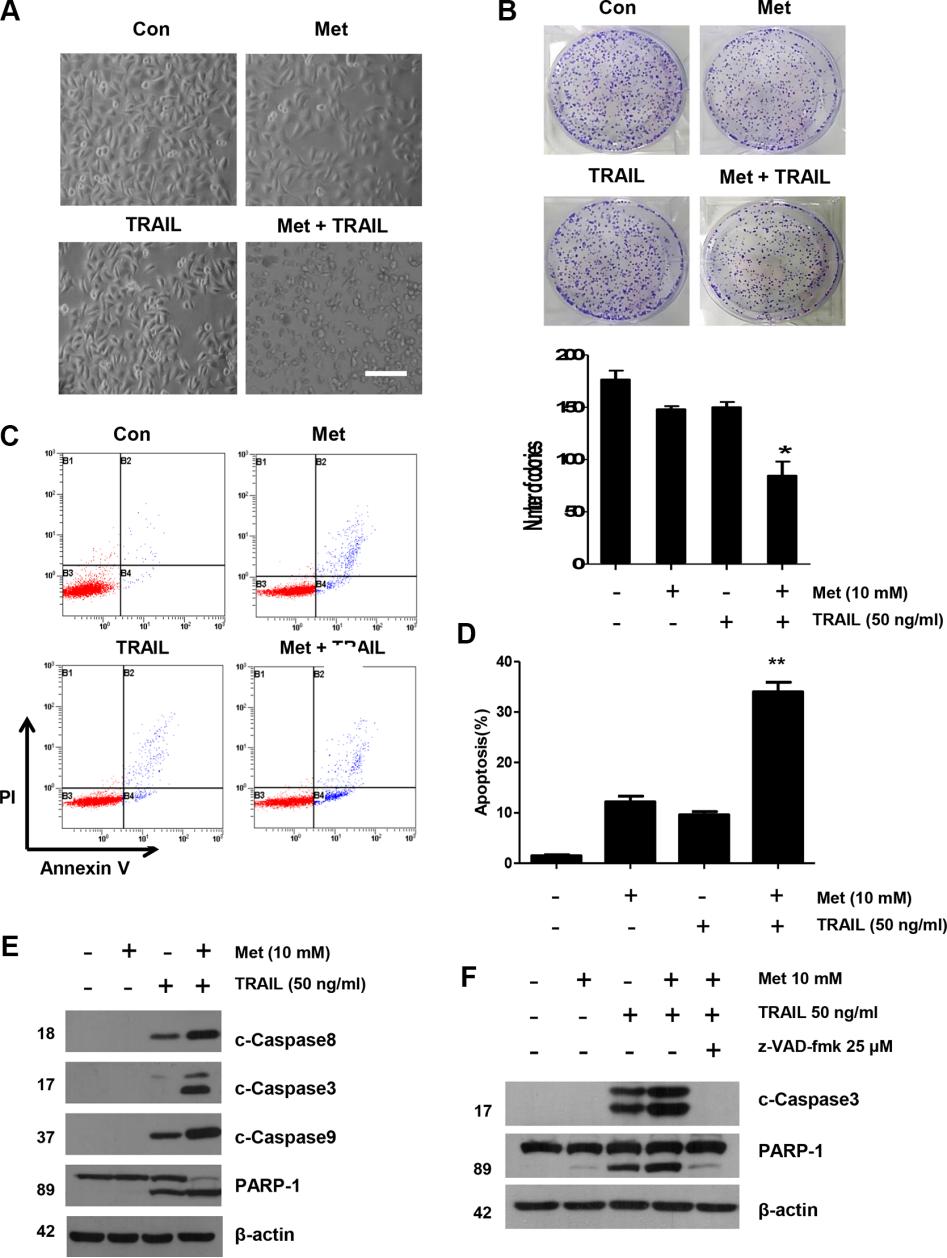
Sensitizing effect of metformin in TRAIL-induced apoptosis in human CRC cells (**A**) Cells were treated with metformin alone, TRAIL alone, or metformin in combination with TRAIL for 24 h. Cell morphology was examined under a light microscope. Scale bar: 100 μm. (**B**) DLD-1 cells plated in 6-well cell culture plates were treated with 10 mM metformin, 50 ng/ml TRAIL, or their combination. The same treatments were repeated every three days. After 12 days, the plates were stained for cell colonies with crystal violet dye, and photographs of colonies taken using a digital camera. (**C** and **D**) The cells were stained with annexin V and propidium iodide (PI), followed by FACS analysis. Error bars represent standard error of the mean (SEM) from three separate experiments. Asterisk * or ** represents a statistically significant difference between treated cells and untreated control cells at *p* < 0.05 or *p* < 0.01, respectively. (**E**) Lysates containing equal amounts of protein (20 μg) were separated by SDS–PAGE and immunoblotted with anti-PARP-1, anti-cleaved caspase-8, anti-cleaved caspase-9, or anti-cleaved caspase-3 antibody. (**F**) Cells were pretreated with 25 μM z-VAD-fmk for 30 min and further treated with metformin (20 h) + TRAIL (4 h) for 24 h. Lysates from cytosolic fractions containing equal amounts of protein (20 μg) were separated by SDS–PAGE and immunoblotted with anti-cleaved caspase-3 and anti-PARP-1 antibody. Actin was used to confirm the equal amount of proteins loaded in each lane.

We further investigated the synergistic interactions between metformin and TRAIL. First, the effect of metformin in combination with TRAIL on DLD-1 cell morphology was examined and photographed under a light microscope. After the application of TRAIL or metformin in combination with TRAIL, as shown in Figure 2A, cell morphology changed significantly when compared to control cells or cells treated with only metformin. Apoptotic cell death with morphological characterstics such as nuclear condensation, cell shrinkage, and blebbing was observed. Cells with morphological changes were counted and statistical significance was calculated (Figure 2A). Additionally, we investigated the long-term effect on clonogenic survival in a cell culture of metformin and TRAIL combination. The metformin and TRAIL combination was much more potent than either agent alone in inhibiting colony formation, in agreement with the apoptosis study. In fact, almost all colonies were eliminated, although metformin or TRAIL alone inhibited the formation and growth of colonies only partially (Figure 2B). Thus, we found that combined treatment with metformin and TRAIL synergistically induced cell death in contrast to metformin or TRAIL alone in CRC cell lines (Figure 1D). We also employed an Annexin V assay, PARP-1 cleavage assay, and cleavage of caspase 8/9 to clarify whether the effect of metformin on TRAIL-mediated cell death was related to apoptosis. As shown in Figure 2C and 2D, we found that, while TRAIL induced apoptosis, metformin increased TRAIL-induced apoptosis in CRC cells. Furthermore, data from biochemical analysis show that, in DLD-1 cells, metformin substantially promoted TRAIL-induced activation of caspase-3, -8, and -9, leading to increased PARP cleavage (Figure 2E). We also used pretreatment with z-VAD-fmk, a pan-caspase inhibitor, to determine that combined treatment with metformin and TRAIL significantly attenuated PARP cleavage (Figure 2F). These results show that increased apoptosis produced by metformin begins through an elevation of the extrinsic and intrinsic pathways.

### Mcl-1 is important for the sensitizing effect of metformin on TRAIL-induced apoptosis of CRC cells

**Figure 3:**
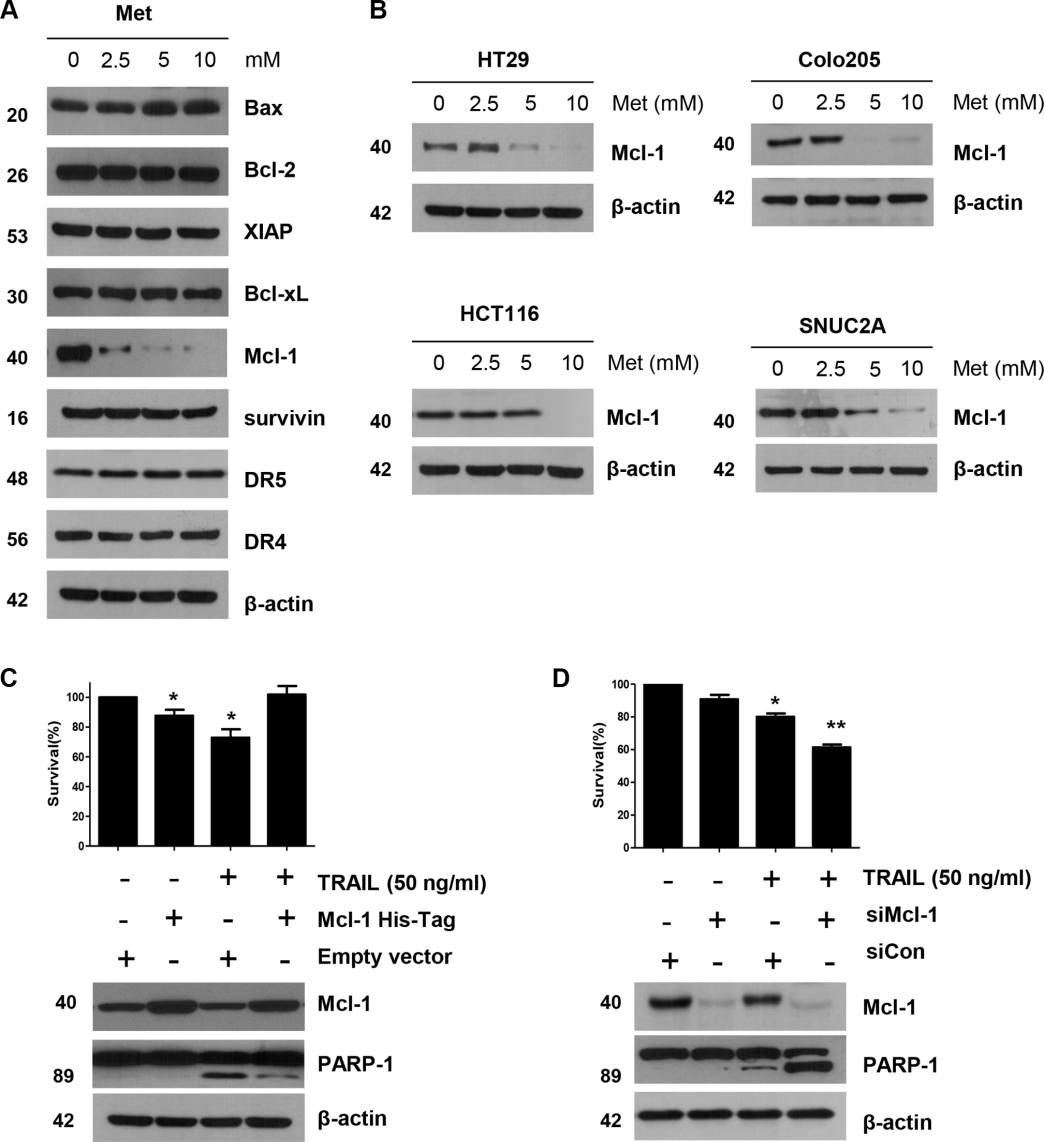
The down-regulation of Mcl-1 by metformin is associated with the induction of TRAIL-mediated apoptosis (**A**) DLD-1 cells were treated with the indicated concentrations of metformin for 20 h. The protein expression levels of survivin, Mcl-1, XIAP, Bcl-2, Bcl-xL, Bax, DR4, DR5, and actin were determined by western blotting. (**B**) HT29, Colo205, HCT116, and SNU C2A cells were treated with indicated metformin doses (0, 2.5, 5, and 10 mM) for 20 h. Cell lysates were analyzed by western blotting using anti-Mcl-1 antibody. (**C**) Empty vector (control) or Mcl-1 stably over-expressing DLD-1 cells (lower panel) were treated with TRAIL for 4 h. Survival was analyzed by trypan blue assay (upper panel). Densitometry analysis of the bands from cleaved PARP-1 or Mcl-1 was performed (lower panel). Error bars represent standard error of the mean (SEM) from three separate experiments. Asterisk * represents a statistically significant difference between TRAIL treated on empty vector transfected cells and TRAIL treated on Mcl-1-His vector transfected cells at *p* < 0.05. (**D**) Mcl-1 was silenced by Mcl-1 siRNA in DLD-1 cells (lower panel). The cells were then treated with TRAIL for 4 h followed by trypan blue (upper panel). Results shown are representative of three independent experiments. Error bars represent standard error of the mean (SEM) from three separate experiments. Asterisk * represents a statistically significant difference between TRAIL + siCon-treated cells and TRAIL + siMcl-1-treated cells at *p* < 0.05.

We investigated whether the sensitizing effect of metformin was similar to that produced by TRAIL when binding to death receptors: activation of the apoptotic signaling pathway via cleavage of caspases and inactivation of anti-apoptotic proteins [6]. We found no change either in the caspase inhibitor protein family members such as survivin, XIAP, and Bcl-XL, or in the Bcl-2 family members such as Bax, and Bcl-2. Death receptors, such as DR4 and DR5, were also unaffected during metformin treatment in DLD-1 cells, as shown in Figure 3A. But, in contrary to these proteins, the level of Mcl-1 decreased in a dose-dependent manner (Figure 3A). This observation was also true in HT29, Colo205, HCT116, and SNU C2A CRC cell lines (Figure 3B). Therefore, we studied the role of Mcl-1 by using recombinant DNA technology. Specifically, we made stable cell using Mcl-1 cDNA expression vector in DLD-1 cells. As shown in Figure 3C, DLD-1 potentiated TRAIL-induced cell death in non-treated cells. However, over-expression of Mcl-1 inhibited DLD-1′s sensitizing effect on TRAIL-induced cell death. Further, knockdown of Mcl-1 by silencing RNA elevated TRAIL-induced cell death (Figure 3D), indicating that, in CRC cells, the sensitizing effect of metformin occurs through down-regulation of Mcl-1 levels.

### Metformin induces Mcl-1 degradation via the ubiquitin-proteasome signaling pathway

**Figure 4:**
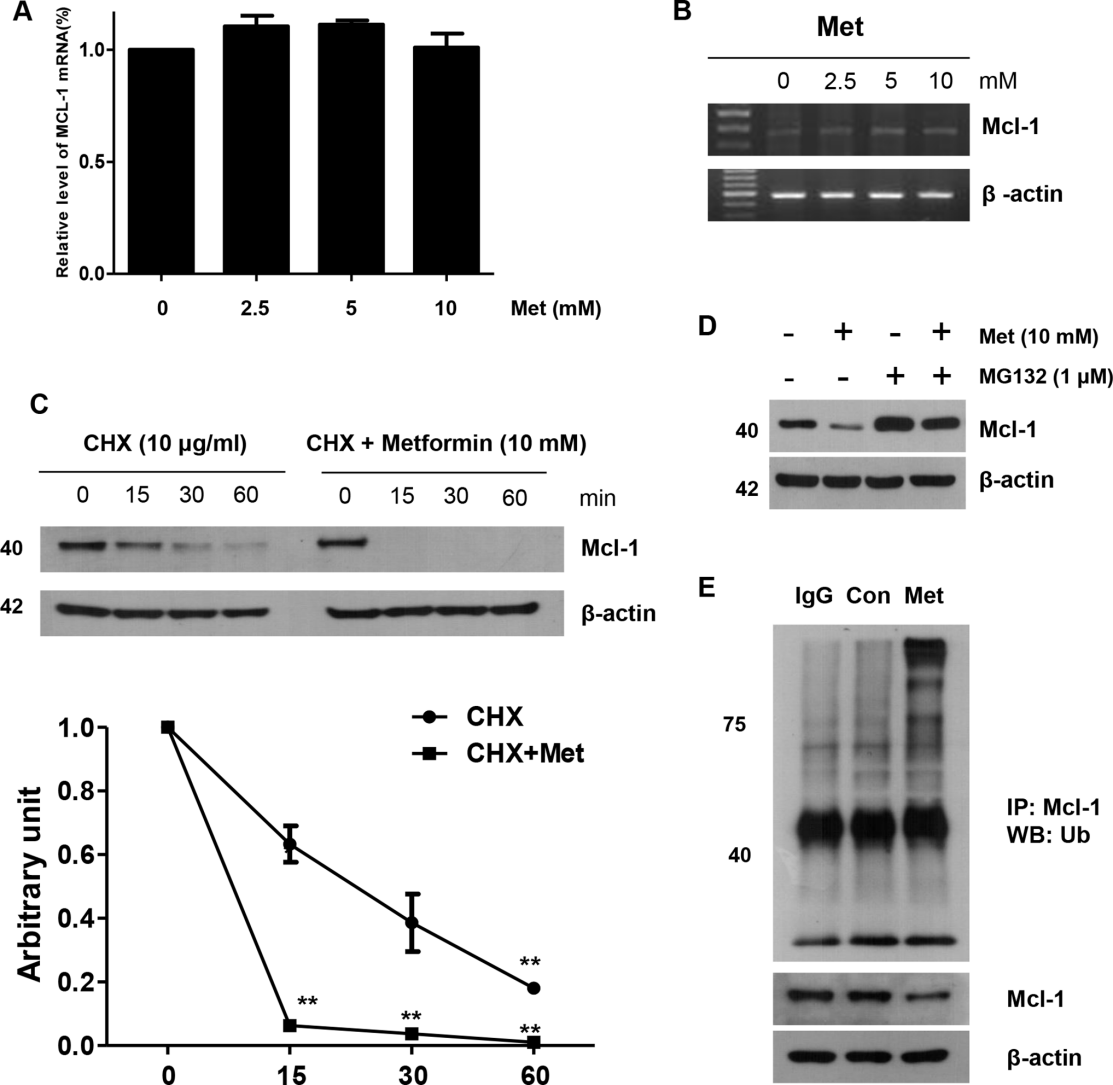
Role of Mcl-1 in the sensitizing function of metformin DLD-1 cells were treated with indicated metformin doses (0, 2.5, 5, and 10 mM) for 20 h. The mRNA expression levels of Mcl-1 and actin were determined by real time -PCR (**A**) and reverse transcriptase -PCR (**B**). (**C**) DLD-1 cells were treated with or without metformin in the presence of cyclohexamide (CHX) (10 μg/ml) for the indicated time periods. The Mcl-1 and actin protein levels were determined by western blotting. Actin expression was used as a loading control. The band intensity of the Mcl-1 protein was measured using the public domain JAVA image-processing program ImageJ. (**D**) Metformin-treated cells were treated with or without MG132 and subjected to western blot analysis using anti-Mcl-1 and anti-actin antibodies. (**E**) Metformin-treated cells were subjected to immunoprecipitation with anti-Ub antibody and immunoblotted for Mcl-1. Error bars represent standard error of the mean (SEM) from three separate experiments. Asterisk * represents a statistically significant difference between metformin-treated cells and untreated control cells at *p* < 0.05.

Next, we investigated whether metformin increased or decreased the level of apoptotic regulatory molecules. As shown in Figure 3A, no significant alterations in levels of the detected apoptotic regulatory proteins were observed, although the expression of Mcl-1 decreased in a dose-dependent manner in the metformin-treated cells within 16 h (Supplementary Figure S1). Therefore, we examined whether metformin modulates Mcl-1 mRNA expression. However, metformin does not affect Mcl-1 mRNA transcription level (Figure 4A and 4B). When DLD-1 cells were or were not exposed to metformin in the presence of 10 μg/ml cyclohexamide (CHX) for the indicated time periods, metformin decreased the Mcl-1 protein stability in DLD-1 cells (Figure 4C). Consistently, in the presence of CHX, the rate of degradation of Mcl-1 was significantly greater in metformin-treated cells than in non-treated ones. These results show that metformin decreases Mcl-1 levels through activation of the ubiquitin-proteasome-dependent pathways. Also, several studies have proven that the degradation of Mcl-1 is generally regulated by the ubiquitin-proteasome pathway [43]. Therefore, we investigated whether metformin also modulates Mcl-1 expression via the ubiquitin-proteasome pathway. First, we investigated the effect of the proteasome inhibitor (MG132) on metformin-induced Mcl-1 degradation. As shown in Figure 4D and 4E, MG132 significantly inhibited the metformin-induced down-regulation of Mcl-1. These results indicate that metformin-induced Mcl-1 degradation is mainly ubiquitin-dependent; however, ubiquitin-independent pathways may also be related to the degradation of Mcl-1. To investigate the pathway of Mcl-1 degradation, we also examined whether Mcl-1 participated in mitogen-activated protein kinase (MAPK) activation in the metformin-treated cells. We found that the MAPK inhibitor (PD098059) did not block Mcl-1 decrease in the metformin-treated cells (Supplementary Figure S1). These data show that the decrease of Mcl-1 plays an important role in metformin-induced TRAIL sensitization.

### Metformin enhances interaction between Mcl-1 and Mule in DLD-1 cells

**Figure 5:**
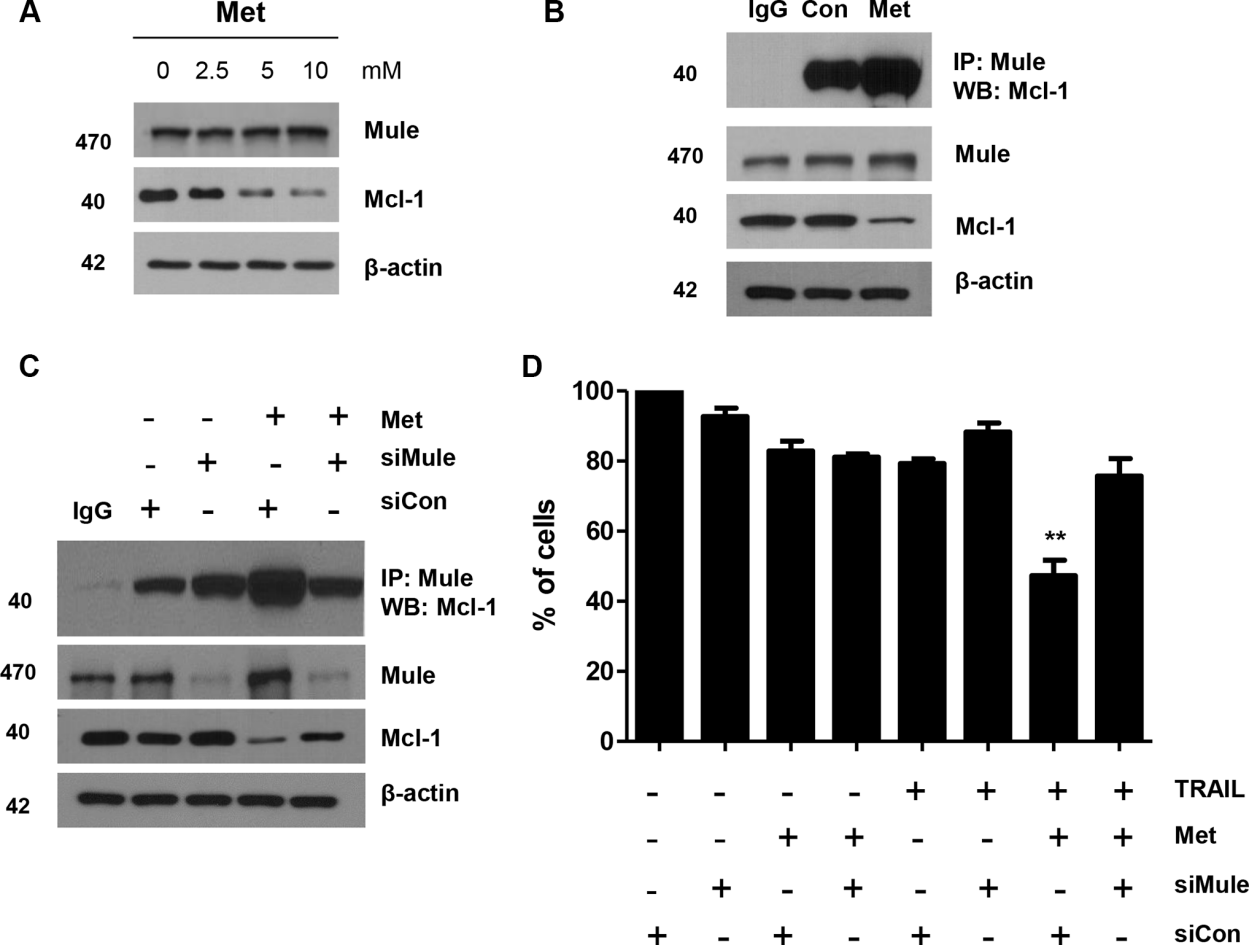
Metformin enhanced Mule/Mcl-1 complex detected in human CRC cells (**A**) DLD-1 cells were treated with indicated metformin doses (0, 2.5, 5, and 10 mM) for 20 h and 50 *μ*g of protein lysates were immunoblotted using anti-Mule and anti-Mcl-1 antibodies. (**B**) DLD-1 cells were treated with metformin for 20 h and immunoprecipitated with anti-Mule antibody or with IgG (16 h) and immunoblotted for Mcl-1. (**C**) Small-inhibitory RNA (siRNA)-mediated inhibition of Mule in DLD-1 cells. Cells were treated with metformin for 20 h and immunoprecipitated with anti-Mule antibody or with IgG (16 h) and immunoblotted for Mcl-1. Western blot analysis showing Mule, Mcl-1 and actin. (**D**) Measurement of apoptotic index in control siRNA and Mule siRNA treated DLD-1 cells by trypan blue assay after metformin and TRAIL treatment. ^**^P < 0.01.

We next investigated facilitators of Mcl-1 ubiquitination and degradation in CRC cells. Mule, also known as E3 ligase, which targets proteins for degradation, has been characterized for Mcl-1. We investigated the expression of Mule by western blot analysis in CRC cells. Mule was universally expressed in DLD-1 cells. While ubiquitination of Mcl-1 decreased in CRC cells, Mule was universally expressed or induced by metformin (data not shown). Therefore, we investigated the interaction between Mcl-1 and Mule by immunoprecipitation assays; immunoprecipitating Mule from metformin-treated cell lysates of DLD-1 cells and immunoblotting with Mcl-1 antibody. We found enhanced Mcl-1 and Mule interaction in metformin-treated cells (Figure 5A and 5B). We confirmed this prolonged interaction between Mule and Mcl-1 in metformin-treated DLD-1 cells (Figure 5C) by immunoprecipitating Mule and blotting for Mcl-1. As a further test, we knocked down Mule expression with silencing RNA. DLD-1 cells treated with either control silencing RNA or Mule silencing RNA were incubated with metformin. The apoptotic index was then analyzed with the trypan blue assay. We found the apoptotic index of these cells significantly decreased through inhibition of Mule levels by silencing RNA treatment (Figure 5D). Our results indicate that decreased ubiquitination and degradation of Mcl-1 in CRC cells may result from the interaction among metformin, Mule, and Mcl-1.

### The E3 ligase Mule is required for proteasomal degradation of Mcl-1 induced by Noxa

**Figure 6:**
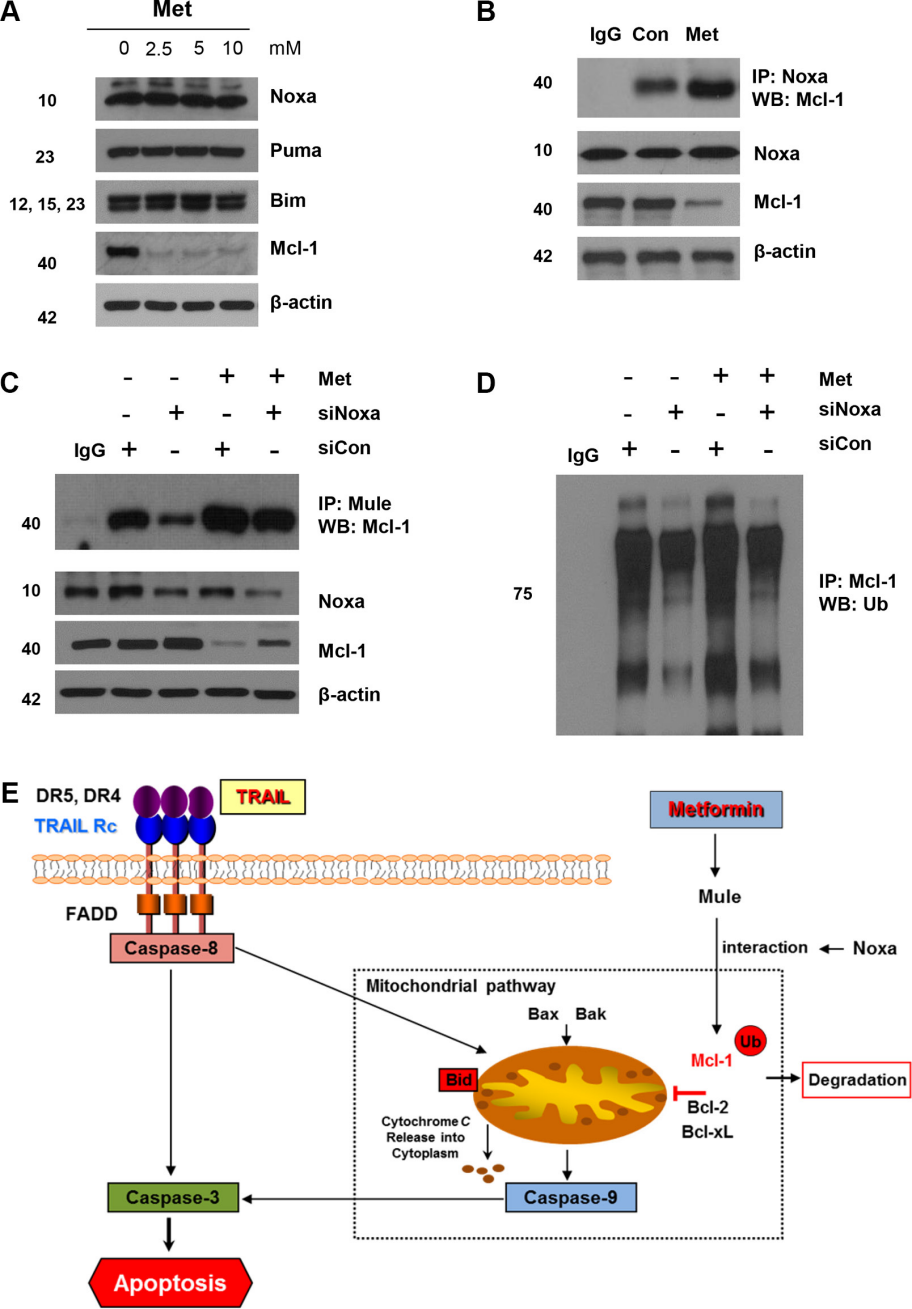
Mule is required for Noxa-induced Mcl-1 degradation following metformin treatment (**A**) DLD-1 cells were treated with indicated metformin doses (0, 2.5, 5, and 10 mM) for 20 h and 50 *μ*g of protein lysates were immunoblotted using anti-Noxa, anti-Puma, anti-Bim, and anti-Mcl-1 antibodies. (**B**) DLD-1 cells were treated with metformin for 20 h and immunoprecipitated with anti-Noxa antibody or with IgG (16 h) and immunoblotted for Mcl-1. (**C** and **D**) Small-inhibitory RNA (siRNA)-mediated inhibition of Mule in DLD-1 cells. Cells were treated with metformin for 20 h and immunoprecipitated with anti-Mule antibody or with IgG (16 h) and immunoblotted for Mcl-1. Western blot analysis showing Mule, Mcl-1 and actin. DLD-1 cells were transfected with a control or Noxa siRNA, and after 40 h the cells were treated with 10 mM metformin for 16 h, and the cell extracts were subjected to immunoprecipitation with an anti-Mcl-1 antibody followed by immunoblot analysis for the indicated proteins. (**E**) Schematic diagram for working model of metformin sensitizing TRAIL-induced apoptosis.

Among the E3 ubiquitin ligases related to Mcl-1 ubiquitination, Mule is particularly involved in the regulation of Mcl-1. Hence, we investigated the interaction between Mcl-1 and Mule in DLD-1 cells with knockdown of Noxa. First, we confirmed that metformin induces increased Bim, Puma, and Noxa expression. As shown in Figure 6A, there was no change during metformin treatment in DLD-1 cells, and in Mcl-1 degradation-related protein members such as Bim, Puma, and Noxa. Even though the Noxa-Mcl-1 interaction was weak during base cases, this interaction increased intensely after the metformin treatment (Figure 6B). We repeated the immunoprecipitation experiments after knockdown of Noxa by silencing RNA to determine the function of Noxa in the Mule/Mcl-1 interaction. In these experiments, the interactions between Mcl-1′s and Mule's were decreased and Mcl-1 polyubiquitination was inhibited by Noxa silencing (Figure 6C). Next, we analyzed the presence of higher molecular weight forms of Mcl-1 to determine if Noxa affects Mcl-1 ubiquitination. We found that knockdown of Noxa triggered a significant decrease in Mcl-1 polyubiquitination (Figure 6D). These data show that Noxa enhances the interaction between Mcl-1 and Mule and as a result, Noxa inhibits degradation by ubiquitin.

## DISCUSSION

In this study, we first demonstrated that combination treatment using metformin and TRAIL synergistically inhibited proliferation of and induced apoptosis in human CRC cell lines, in addition to reducing the expression of Mcl-1, p-JAK2, and p-STAT3 (Supplementary Figure S1). Compared to the individual agents, the metformin and TRAIL combination significantly inhibited colony formation in and migration of CRC cells, as well as significantly decreased Mcl-1 and Bcl-2 (anti-apoptotic proteins), and increased Bim and Puma (pro-apoptotic proteins) (Supplementary Figure S1).

Previous studies have demonstrated that Mcl-1 protein has a critical role in the anti-apoptotic system in CRC cells. Lee et al. [44] showed that tumor specimens overexpressed Mcl-1 in CRC patients. We found that metformin increased Mcl-1 degradation via ubiquitin and induction of cell death by downregulating STAT3 in CRC cells. In addition, higher apoptosis induction was observed when Mcl-1 was directly knocked down by silencing RNA for Mcl-1 or indirectly by inhibiting pERK1/2 (Figure 4B). Our data are consistent with those of a number of reports, which show that drugs such as bortezomib, Obatclax (a Bcl-2 inhibitor), and irinotecan downregulated Mcl-1 to induce apoptosis in CRC cells [45–47]. Mcl-1 is also an anti-apoptotic protein known to be degraded via the ubiquitination-proteasome pathway [48]. Glycogen synthase kinase 3 (GSK3β) is a key regulator in tumorigenesis and mediates phosphorylation of Mcl-1 at Ser159 [49, 50]. As a result, GSK3β can be associated with β-transducin repeat-containing protein (β-TrCP) or F-box/WD repeat-containing protein 7 (FBXW7) (E3-ubiquitin ligases), resulting in Mcl-1 degradation [49, 51, 52]. Therefore, GSK3β plays a critical role in the negative regulation of Mcl-1 stability. We showed that metformin did not inhibit GSK3β phosphorylation in a dose-dependent manner (Supplementary Figure S1). In the present study, we showed that metformin decreased Mcl-1 levels, which may indicate a major event in increasing the synergism with TRAIL in dying CRC cells. This event likely occurs at the post-translational level via reaction of enzymes that regulate Mcl-1 [53]. The data presented here also indicate that metformin regulates Mcl-1 expression by stimulating proteasome-mediated degradation. Furthermore, the decrease of Mcl-1 occurred without regard to change in Mcl-1 mRNA transcriptional levels (Figure 4A and 4B). This finding is important because Mcl-1 levels are related to initial tumor relapse and shorter survival rate in patients with CRC [44]. In addition, high levels of cellular antiapoptotic proteins have been hypothesized to be due to TRAIL resistance in numerous types of cancer. Notably, increased Mcl-1 levels have been shown to inhibit TRAIL-induced CRC cell death, while a decrease in Mcl-1 increased TRAIL-induced apoptosis in CRC cells [54]. This suggests that overexpression of Mcl-1 could reduce the effect of apoptotic stimuli such as TRAIL in inducing cell death, while decreases in Mcl-1 have been associated with enhanced cytotoxicity of TRAIL [55]. This finding is consistent with other reports of inhibition of Mcl-1 by using metformin, further emphasizing the crucial role of Mcl-1 in metformin-induced apoptosis.

Overexpression of Mcl-1 has been found to be an indicator of tumor progression and poor outcomes in numerous human tumors [56–59]. To our knowledge, this is the first time that metformin has been shown to decrease Mcl-1 expression in CRC cells. Even though Mcl-1 potentially could be controlled at multiple levels [60, 61], metformin did not affect the transcriptional level, protein stability, or degradation of Mcl-1 in CRC cells, indicating that the metformin-induced decrease of Mcl-1 protein was not because of transcriptional or post-translational regulation.

We hypothesized that metformin inhibits *de novo* protein synthesis and we studied mechanisms that increase the stability of Mcl-1 in CRC cells. Notably, we found that stable binding of Mcl-1 with the E3 ligase, Mule, may be one of the prominent mechanisms that increase the stability of Mcl-1 in CRC cells. We believe that this is the first report of a transient association between Mule and Mcl-1 in CRC cells. Mule is a novel ubiquitin, which can specifically interact with Mcl-1 [43, 62]. We detected a stable prolonged association between Mcl-1 and Mule in metformin-treated DLD-1 cells, in which Mcl-1 undergoes prominent ubiquitination and degradation. We found an induced ubiquitination of Mcl-1 as well as a stable association of Mcl-1 with its specific E3 ligase, Mule, in CRC cells treated with metformin. Metformin induced down-regulation of Mcl-1 protein expression at the post-translational level (Figure 3B). Furthermore, Mule has been determined to regulate the degradation of Mcl-1 proteins. Mule has been known to be a multi-adaptor protein and an E3 ligase. Gomes-Bouqie *et al*. reported that Mule is responsible for degrading Mcl-1 by proteasomes in prostate cancer cells [63]. In this study, metformin attenuated cell death when Mule was downregulated by Mule siRNA (Figure 5C). These data show that metformin increased the stability of Mule in CRC cells. We also found that metformin counteracts Mcl-1 by inhibiting Mcl-1 for ubiquitin proteasome-induced degradation in the CRC cells. Noxa also appears to play a major regulator of Mcl-1 stability. Noxa has been presented to negatively regulate Mcl-1 [64], potentially by increasing the binding of Mule (E3 ligase) and decreasing the binding of the USP9X (deubiquitinase) to Mcl-1 [63]. This is confirmed by our results that Mcl-1 interacts with Noxa in control cells and that the knockdown of Noxa by silencing RNA elevated Mcl-1 protein (Figure 6C). In addition, metformin quickly replaces Noxa from Mcl-1, allowing Mule binding and degradation by the ubiquitin-proteasome pathway to occur (Figure 6D). In accordance with a previous report, our data show that Noxa protein was not altered by metformin [65].

*In vitro*, the Mcl-1 protein was decreased by either metformin or TRAIL, and combination of the two drugs further decreased Mcl-1 protein levels. As Mcl-1 is strongly increased by proteasome degradation, we studied the effects of metformin and TRAIL with and without of MG132, a proteasome inhibitor. Our results showed that metformin and TRAIL did not change Mcl-1 with MG132, indicating that metformin and TRAIL induced proteasome degradation of Mcl-1. These findings imply that metformin and TRAIL regulate Mcl-1 protein partially through proteasome degradation.

Additionally, AMPK has been reported to be a major regulator of metformin in various cancer types. In the present study, although it is possible that AMPK could be activated by metformin, which would be inconsistent with previous studies, the anti-proliferation effect of metformin does not require AMPK activation. When AMPKα was knocked down by silencing RNA, the survival rates of the cells treated with metformin, TRAIL, or the combination were not statistically significantly different (data not shown). In addition, the decrease of Mcl-1 by metformin was observed even in the absence of AMPKα, showing that downregulation of Mcl-1 by metformin is independent of AMPK activation.

STAT3 is a main modulator in inflammation-mediated tumorigenesis, including CRC, by promoting tumor cell proliferation and survival. We found that metformin significantly suppressed the phosphorylation of STAT3 in CRC cells. This observation is consistent with the previous findings that metformin inhibited the transcriptional activation of STAT3 in various cancer cell lines [66–68]. STAT3 is also known to modulate Mcl-1 translation, which supports STAT3-dependent cell growth and proliferation. In our study, however, decreasing STAT3 levels produced no change in the cells' sensitivity cell to metformin. Moreover, Mcl-1 protein did not change in STAT3-silenced cells. In our study, we also detected a down-regulation of STAT3 in metformin-treated cells (data not shown). Our previous studies have reported that inhibition of STAT3 by S31-201 (STAT3 inhibitor) or silencing RNA led to a significant decrease in the level of Mcl-1 and enhanced HSP90 inhibitor (NVP-AUY922)-induced apoptosis in CRC cells [54]. Therefore, inhibition of STAT3 may be an effective medical treatment to sensitize cancer cell to apoptotic stimuli such as radiation or anticancer drugs. Furthermore, a JAK/STAT3-signaled decrease of Mcl-1 protein is considered to be a crucial factor responsible for metformin-induced sensitization to TRAIL in CRC cells. Thus, we propose that metformin may cause a decrease of Mcl-1 through the JAK/STAT3 signal of its regulation. Here, we determined that metformin inhibited the phosphorylation of JAK2 and STAT3, but could not effectively decrease the Mcl-1 level (Supplementary Figure S1). However, this down-regulation of STAT3 had no effect on Mcl-1 expression or TRAIL sensitization in metformin-treated cells (data not shown). Hence, these data show that the decrease of Mcl-1 by metformin is independent of the down-regulation of JAK/STAT3 expression in CRC cells.

Last, it has been found in breast cancer cells that ERK1/2 can phosphorylate and stabilize Mcl-1 [69]. We found ERK1/2 and its active form pERK1/2 to be upregulated in all the CRC cells examined; their levels were downregulated after metformin treatment, pointing to increased stability of these proteins (Supplementary Figure S1). Inactivating pERK1/2 with the MEK inhibitor, U0126, promoted decline of Mcl-1 levels in CRC cells (data not shown).

In conclusion, our findings show that the novel mechanism of cell death induced by metformin involves TRAIL sensitization and degradation of Mcl-1, important for an understanding of its role in the pathophysiology, diagnosis, and ways to treat cancer. Finally, these data are the first to indicate that metformin suppresses cell proliferation and enhances apoptosis when used in combination with TRAIL in human CRC cells, possibly through Noxa-mediated increase in Mcl-1 degradation via the ubiquitin-proteasome pathway.

## MATERIALS AND METHODS

### Cell culture

The human colon carcinoma cell lines DLD-1, HT29, Colo205 and HCT116 were obtained from the American Type Culture Collection (ATCC, Manassas, VA, USA) and maintained according to the ATCC's instructions. Primary cultures of human normal colon cells (FHC) and their corresponding growth medium (DMEM:F12) were purchased from ATCC (Manassas, VA, USA). All cell lines were grown in RPMI 1640 supplemented with 10 % FBS and l-glutamine and grown in a 37°C humidified chamber 5% CO_2_.

### Reagents and antibodies

Metformin was purchased from Wako (Richmond, VA, USA). TRAIL (Recombinant human) was purchased from Millipore (Millipore, Darmstadt, Germany.) Protein G PLUS-Agarose, Anti-Bax, anti-Bcl-2, anti-Mcl-1(IP), anti-Ub and anti-Bcl-xL were purchased from Santa Cruz Biotechnology (Santa Cruz, CA, USA). Anti-XIAP, anti-phospho ERK, anti-ERK, anti-phospho JAK2, anti-JAK2, anti-phospho AMPK, anti-AMPK, anti-phospho mTOR, anti-mTOR, anti-phospho AKT, anti-AKT, anti-phsopho GSK3β, anti-GSK3β, anti-Noxa, anti-Puma, anti-Bim, anti-phospho Mcl-1, anti-Mcl-1(WB), anti-cleaved caspase-3, anti-cleaved caspase-8, anti-cleaved caspase-9, anti-phospho STAT3, anti-STAT3, and anti-PARP-1 were purchased from Cell Signaling (Beverly, MA, USA). Anti-actin antibody was purchased from Sigma (Sigma, St. Louis, MO). Anti-Mule antibody was purchased from Abcam (Cat. No. ab70161). For the secondary antibodies, anti-mouse-IgG-HRP and anti-rabbit-IgG-HRP were purchased from Cell Signaling (Beverly, MA, USA).

### Small interfering RNA (siRNA)

Mcl-1 siRNA (Cat. No. SC-35877), Noxa siRNA (Cat. No. SC-37305), Mule siRNA (Cat. No. SC-61758) and negative control siRNA (Cat. No. SC-37007) were obtained from SantaCruz Biotechnology (Santa Cruz, CA, USA). Cells were transfected with siRNA oligonucleotides using Lipofectamine RNAi Max reagents (Invitrogen) according to the manufacturer's introductions. After 24 h of transfection, cells were treated with TRAIL and metformin for further analysis.

### Transient and stable transfection

Cells were transfected with human Mcl-1 tagged with Flag in pCDNA3.1 vector or the corresponding empty vector (pCDNA). Mcl-1 overexpression plasmid pTOPO-Mcl-1 (Plasmid No 21605) was purchased from Addgene. Cells were selected with 1 mg/ml G418 for 2 weeks and five clones were pooled and then maintained in 500 μg/ml G418.

### Survival assay

Cells were grown in tissue culture-coated 96-well plates and treated as described in Results. Cells were then treated with 3-(4,5-dimethylthiazol-2-yl)-2,5-diphenyltetrazolium bromide (MTT) assay (Roche Molecular Biochemical, Indianapolis, IN, USA) for 3h at 37°C in an atmosphere of 5% CO2. Absorbance at 450 nm was determined using an enzyme-linked immunosorbent assay plate reader. The total number of cells in each well was counted under a microscope. Then, cells were dissociated and stained with trypan blue (Amresco, Inc., Solon, OH, USA).

### Colony formation assay

Colony formation on plate was conducted in 6-well cell culture plates as previously described [70].

### Apoptosis assay (flow cytometry)

The translocation of phosphatidylserine, one of the markers of apoptosis, from the inner to the outer leaflet of plasma membrane was detected by binding of allophycocyanin (APC)-conjugated annexin V. Briefly, DLD-1cells untreated or treated with metformin, TRAIL, or a combination of the two agents was resuspended for 24 h in the binding buffer provided in the Annexin V-FITC Apoptosis Detection Kit (BioBud, Cat. LS-02-100). Cells were mixed with 1.25 μL Annexin V-FITC reagent and incubated for 30 min at room temperature in the dark. The staining was terminated and cells were immediately analyzed by flow cytometry.

### Western blotting

Western blotting was carried out as previously described [54]. Immunoreactive proteins were visualized by the chemiluminescence protocol (ECL, Dogen, Seoul, Korea).

### RT-PCR analysis

Total RNA extraction was performed using the TRIzol reagent (Life Technologies, Rockville, MD, USA), according to the manufacturer's instructions. Amplification of transcripts was performed by reverse transcriptase polymerase chain reaction kit (Life Technologies). PCR amplification was performed using the following primers: Mcl-1, forward: 5′- GCG ACT GGC AAA GCT TGG CCT CAA-3′, reverse: 5′- GTT ACA GCT TGG ATC CCA ACT GCA -3′.

### Real time PCR

Total RNA was extracted by using TRIzol reagent (Life Technologies). Amplification of transcripts was performed by reverse transcriptase polymerase chain reaction kit (Life Technologies). RT-PCR was performed on an Applied Biosystems 9700 RT-PCR using gene-specific oligonucleotide primer for Taqman probes (Applied Biosystems). Taqman probes were as follows: GAPDH (Hs99999905_m1), Mcl-1 (HS01050896_m1). For expression of mRNA, gene expression was normalized by the GAPDH.

### Co-immunoprecipitation

Cells were washed with ice-cold PBS and incubated on ice for 5 min with 300 μl lysis buffer (Cell Signaling, Cat. No. 9803) (1 mM PMSF, protease inhibitor, and phosphatase inhibitor). The cells were scrape-harvested, and cellular debris were removed by centrifugation for 5 min at 15,000 rpm at 4°C, and the concentration of protein was determined by BCA assay (Thermo Scientific). Cell supernatants were incubated with primary antibody for overnight at 4°C, followed by addition of 50 μl protein G agarose beads (50% slurry) for 1 h at 4°C. Immunoprecipitates were washed five times with ice-cold lysis buffer, separated by centrifugation for 30 s at 10,000 rpm, and then heated with 2x sample buffer for electrophoresis and Western blot analysis.

### Statistical analysis

Statistical analysis was carried out using Graphpad InStat 6 software (GraphPad Software, Inc., San Diego, CA, USA). The results were expressed as the mean of arbitrary values ± SEM. All results were evaluated using an unpaired Student's *t* test, where a *p*-value of less than 0.05 was considered significant.

## SUPPLEMENTARY MATERIALS FIGURE



## Abbreviations

DR5: death receptor-5
PARP: poly (ADP-ribose) polymerase
PBS: phosphate-buffered saline solution
PI: propidium iodide
SDS-PAGE: sodium dodecyl sulfate polyacrylamide gel electrophoresis
TNF: tumor necrosis factor
TRAIL: tumor necrosis factor-related apoptosis-inducing ligand.
