# Supplementary material for: Metformin enhances TRAIL-induced apoptosis by Mcl-1 degradation via Mule in colorectal cancer cells
Source: Oncotarget. 2016 Aug 9;7(37):59503–18. doi: 10.18632/oncotarget.11147 (PMC5312327; doi:10.18632/oncotarget.11147)
Supplement: Supplementary file 1 [file oncotarget-07-59503-s001.pdf]

Metformin enhances TRAIL-induced apoptosis by Mcl-1 degradation via Mule in colorectal cancer cells

Supplementary Materials

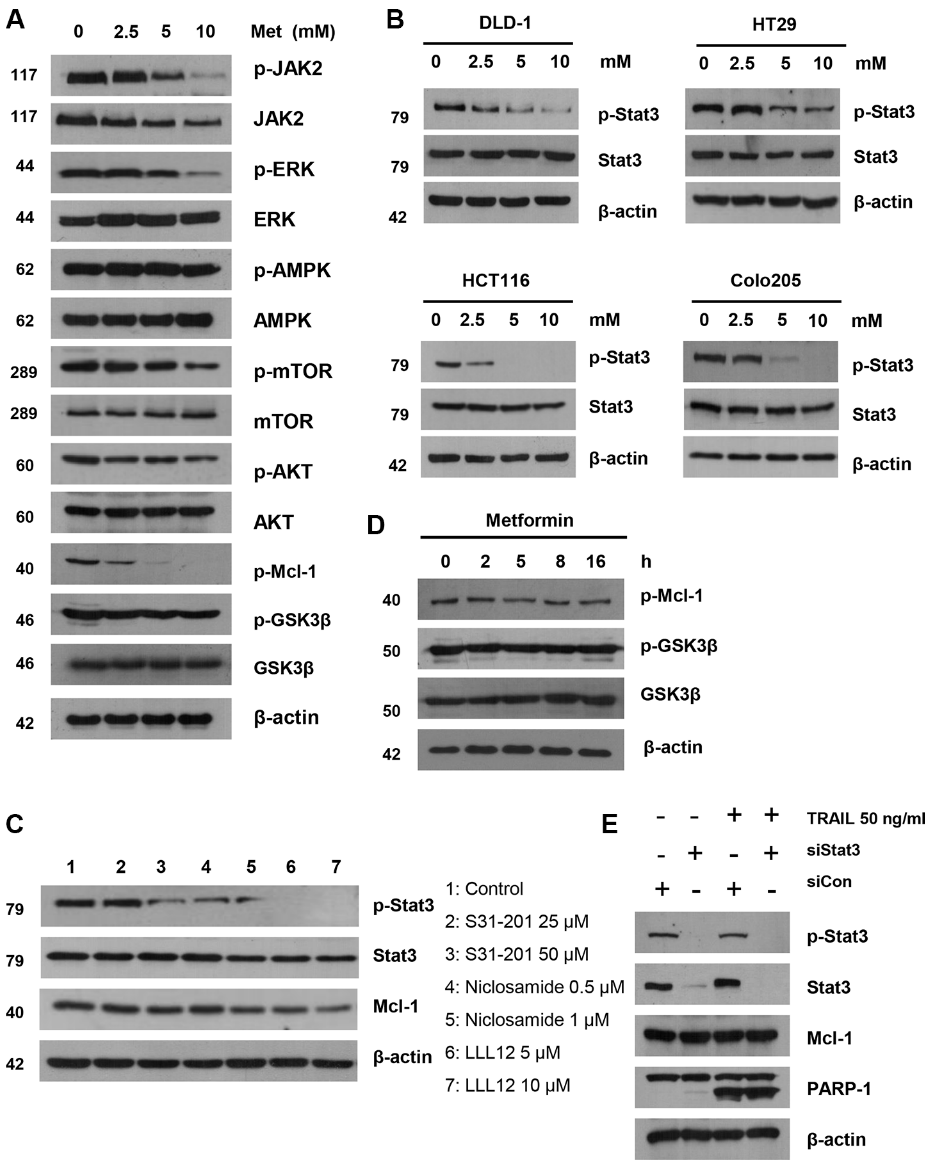

Supplementary Figure S1: (**A**) DLD-1 cells were treated with indicated metformin doses (0, 2.5, 5, and 10 mM) for 20 h. Western blotting analysis was done by using indicated antibodies (p-JAK2, JAK2, p-ERK, ERK, p-AMPK, AMPK, p-mTOR, mTOR, p-AKT, AKT, p-Mcl-1, p-GSK3β, GSK3β). (**B**) DLD-1, HT29, HCT116, and Colo205 cells were treated with indicated metformin doses (0, 2.5, 5, and 10 μM) for 20 h. Cell lysates were analyzed by western blotting using anti-phospho-Stat3 antibody. (**C**) DLD-1 cells were treated with S31-201 (25 and 50 μM), niclosamide (0.5 and 1 μM), or LLL12 (5 and 10 μM) for 20 h. Lysates containing equal amounts of protein were separated by SDS–PAGE and immunoblotted with anti-phospho-STAT3, anti-STAT3, or anti-Mcl-1 antibody. Actin was shown as an internal standard. (**D**) DLD-1 cells were treated with 10 mM metformin for various times (0–16 h). Lysates containing equal amounts of protein (20 μg) were separated by SDS–PAGE and immunoblotted with anti-phospho-Mcl-1, anti-phospho-GSK3β, anti-GSK3β antibody. Actin was shown as an internal standard. (**E**) STAT3 was silenced by STAT3 siRNA in DLD-1 cells. Results shown are representative of three independent experiments.
